# Effect of acute green tea extract ingestion on fat oxidation during exercise in women

**DOI:** 10.1186/1550-2783-11-S1-P15

**Published:** 2014-12-01

**Authors:** Harriet Lloyd, Conrad P Earnest

**Affiliations:** 1 University of Bath Sports, Bath, United Kingdom

## Background

Green tea and green tea extract (GTE) consumption is associated with health, exercise performance, fat oxidation and weight loss owing to studies reporting positive effects accompanying chronic supplementation (>30 d). While the overall literature surrounding GTE is equivocal, little data exists examining the effects of acute supplementation prior to exercise (< 24 hr). The primary aim of our current study was to investigate the effects of acute decaffeinated GTE ingestion on fat oxidation rates during moderate-intensity exercise in physically active females.

## Methods

We randomized 10 physically active females (mean ± SD: age: 20 ± 1.2 y; weight: 65.7 ± 6.1 kg; body mass index: 23.8 ± 2.2 kg/m2; VO2max: 51.4 ± 6.2 ml kg-1 min-1) to participate in our study. Prior to treatment, the participants volunteered to perform a VO2max test in order to establish their exercise testing intensity. Subsequently, we randomly assigned to the double-blind, counter-balanced, cross-over treatments of encapsulated, decaffeinated, GTE or a colour matched corn flour placebo (PLA) in two doses; 2 capsules with dinner the night before, 2 capsules with breakfast the morning of testing. Treatment capsules contained 340 mg polyphenols and 85 mg EGCG. Testing consisted of having participants initiate riding on bicycle ergometer for 45 min at a workload associated with 65% VO2max. Respiratory gas measurements were collected at rest and in 3 min epochs, 5 min after starting exercise and every 15 min during exercise, in order to calculate fat and carbohydrate oxidation and total energy expenditure. Secondary measures included heart rate (HR) and ratings of perceived of exertion (RPE) during exercise testing. Overall carbohydrate and fat oxidation were analysed using an integrated area-under-the-curve (AUC) from rest through the completion of exercise. A paired-sample t-test compared AUC for fat oxidation and the total, and relative substrate contribution to, energy expenditure at rest and during exercise. Potential differences in fat oxidation, carbohydrate oxidation, RER, HR and RPE between treatment groups were examined at each time point including 0 (rest), 5, 15, 30 and 45 minutes into steady-state cycling using a 2-factor (treatment x time) repeated measures analysis of variance (ANOVA). Data are presented as mean ± SD or 95% confidence intervals (CI) when appropriate. Significance was set at P < 0.05. Consent to publish the results was obtained from all participants.

## Results

Overall, the acute ingestion of each respective supplement had no significant effect for GTE (20.8 g/min, 95 % CI, 12.2, 24.0) or PLA and 18.19 g/min, 95% CI, 12.2, 24.0). Moreover, the relative contribution of fat to total energy expenditure at rest (42% PLA; 36% GTE) and during exercise (28% PLA; 23% GTE) was not significantly different between trials. No further effects were noted for total energy expenditure, and carbohydrate and fat oxidation, RER, HR and RPE at each respective time point.

## Conclusions

In contrast to previous reports, the acute ingestion of decaffeinated GTE did not significantly alter whole-body fat oxidation rates during exercise in physically active females. However, the response to GTE was highly variable between individuals; thus, more research is required to investigate potential moderators of the effects of GTE.

